# AI-driven decision making for intravascular device selection in aortic disease. Current insights and prospects

**DOI:** 10.3389/fcvm.2025.1585299

**Published:** 2025-11-20

**Authors:** Rasit Dinc, Nurittin Ardic

**Affiliations:** 1 INVAMED Medical Innovation Institute, New York, NY, United States; 2 Med-International UK Health Agency Ltd., Leicestershire, United Kingdom

**Keywords:** aortic aneurysm, artificial intelligence, endovascular repair, medical image segmentation, intravascular device selection

## Abstract

Abdominal and thoracic aortic repairs increasingly rely on endovascular solutions, but device selection in anatomically complex cases remains prone to error due to measurement variability, tortuosity, short/angulated necks, and heterogeneous post-EVAR evolution. This article focuses on artificial intelligence (AI) tools that support intravascular device selection and planning, particularly in abdominal and thoracic aortic aneurysms, and type B dissection scenarios where endovascular repair (EVAR/TEVAR) is applicable. We synthesize evidence on (i) automated centerline extraction and 3D measurements that standardize sizing; (ii) risk models that predict inadequate sealing or endoleakage to guide oversizing and landing zone strategy; and (iii) procedural environment “augmented intelligence” maps and extended reality modules that operationalize device selections in the laboratory. We summarize commercial and research-level systems, clinical readiness, and regulatory status, and outline validation, explainability, and bias considerations. While current evidence-based workflows achieve excellent results, targeted AI components reduce variability and can support consistent device decisions across complex anatomies. Prospective, multicenter validation is needed before routine implementation; for now, AI should be viewed as a complement, rather than a replacement, to established EVAR/TEVAR planning and oversight.

## 1 Introduction

Aortic diseases, encompass a spectrum of life-threatening conditions, primarily abdominal and thoracic aortic aneurysms (AAA and TAA) and aortic dissection (AD). Although these conditions vary in pathophysiological and clinical presentation, they share a common risk of high morbidity and mortality when left untreated (1, 2).

While treatment can be lifesaving, there is no established pharmacologic treatment for most aortic diseases (3). Open or endovascular surgical repair is the mainstay of intervention. For suitable patients, endovascular approaches such as EVAR (for AAA) and TEVAR (for TAA or complicated AD) offer minimally invasive alternatives to open surgery (4, 5). Current treatment of aortic disease is based on well-established guidelines that have yielded excellent results. EVAR achieves mortality rates in the low single-digit percentage range, and current imaging protocols provide rapid and accurate diagnosis in the majority of cases (6, 7). However, the rates of morbidity and mortality vary among the studies. Caradu et al. (8) reported that complications such as device migration or endoleak occur in up to 25% of patients undergoing EVAR.

Accurate device selection and measurement of aortic dimensions are crucial for the success of endovascular repair. The computed tomography angiography (CTA) imaging has also an essential role in post-procedural monitoring (9–11). Despite these advantages, these procedures can be complex and time-consuming, especially in cases with tortuous anatomy or dissection. Additionally, traditional planning relies heavily on clinician expertise and manual image interpretation, which leads to variability and can increase the risk of complications such as endoleaks or device migration (8, 12). These risks underscore the need for precise preoperative planning and meticulous postprocedural imaging surveillance, while addressing resource-intensive and standardization challenges.

Despite guideline-driven workflows, device selection remains challenging due to patient-specific anatomy (short/angulated necks, thrombus/calcification, iliac access), interobserver variability in 3D measurements, and post-EVAR risks such as seal failure, endoleak, and sac enlargement or migration. These factors lead to reinterventions and variability across centers, even when CTA protocols are standardized (13, 14). Therefore, the specific goal of this review is to examine AI modules that directly impact device selection steps (from automated centerline and sizing to risk-based oversizing and landing zone strategy) and to clarify how such tools help (or potentially could help) clinicians make better device decisions rather than providing generic analyses independent of procedural choices. Advances in computational technologies, particularly artificial intelligence (AI), have created new opportunities to improve clinical decision-making in vascular medicine. AI is a general term that includes machine learning (ML), which allows systems to learn from data, and deep learning (DL), a subset of AI that uses neural networks to process complex imaging or clinical inputs. These tools have shown early promise in risk prediction, anatomical segmentation, and image interpretation. In the context of aortic disease, AI has the potential to support more precise preoperative planning, improved procedural simulations, and personalized surveillance strategies (13–16).

This article provides a narrative overview of emerging applications of AI in the treatment of aortic diseases, focusing specifically on their potential to assist in intravascular device selection and procedure planning in endovascular repair. The discussion addresses anatomical challenges, imaging interpretation, clinical risk stratification, and future research directions. Given current limitations in clinical evidence, this review emphasizes the prospective value of AI rather than asserting definitive clinical superiority.

## 2 Aortic pathologies and management

### 2.1 Pathology-specific background and treatment overview

Cardiovascular diseases remain the leading cause of death globally, with aortic pathologies representing some of the most acute and high-risk conditions (17). The most clinically significant of these are AAA, TAA, and AD (18, 19). These conditions differ in etiology, natural history, imaging requirements, and treatment modalities and should be considered separately (5, 20).

**Figure 1:**
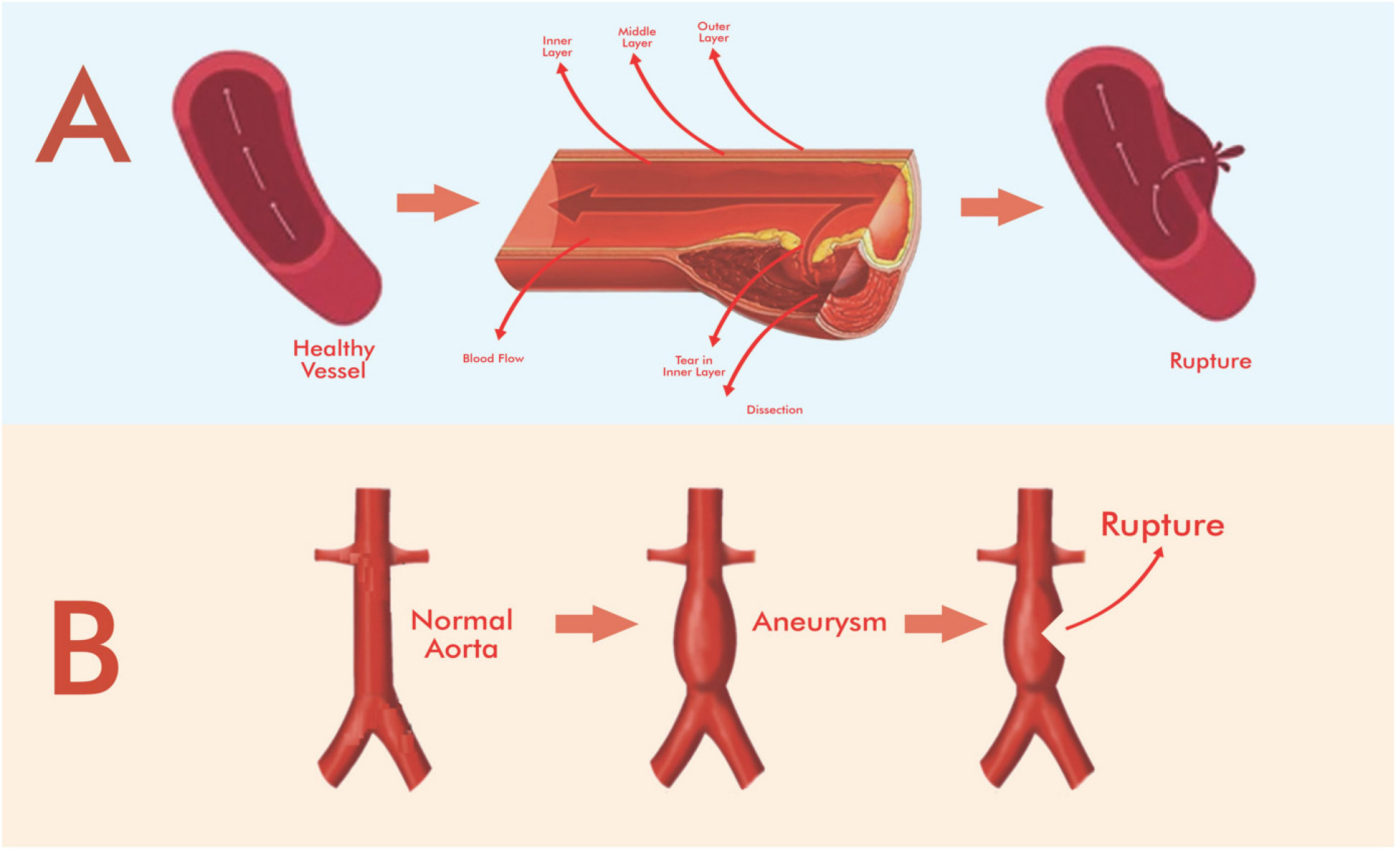
Simplified schematic representation of the stages of aortic aneurysm and dissection and the effect of stenting on blood flow. **(A)** Aortic dissection, **(B)** aortic aneurysm. Adapted from “Schematic illustration of abdominal aortic aneurysms (AAA) pathogenesis and its macrophage polarization therapy” by Rasit Dinc (Taiwan Society of Cardiology (Acta Cardiologica Sinica). Drawing with Adobe Creative Suite Package [(Illustrator, version 28.7.1 and Photoshop, version 25.12)].

AAA and TAA are characterized by progressive dilation of the vessel wall, usually due to a combination of genetic factors, atherosclerosis, and degenerative changes in the media. AAAs are most commonly seen beneath the renal arteries, while TAAs affect the ascending or descending thoracic aorta (5, 20). If untreated, aneurysmal rupture has a mortality rate of up to 80%, especially in abdominally located cases (18). AD, in contrast, causes a tear in the intimal layer of the aorta, allowing blood to enter the medial layer and creating a false lumen (1, 21) (Figure 1). Dissections are classified as Stanford type A (ascending aorta) and type B (descending aorta) and present acutely. Without prompt diagnosis and treatment, mortality in type A dissections can approach 50% within the first 48 h (1, 19).

Standard treatment for AAA and selected cases of TAA includes open surgical repair or EVAR for AAA and TEVAR for TAA (4, 5). EVAR is preferred in elective AAA cases due to reduced perioperative risk. However, anatomic suitability must be confirmed with preoperative imaging to avoid complications such as endoleaks or device migration (8, 12). Not all aneurysms, especially those with short or angled necks, are suitable for endovascular techniques. In contrast, treatment of AD varies by type: Type A dissections usually require urgent open surgery, while uncomplicated Type B dissections can be managed medically, and TEVAR is reserved for complicated cases (1, 2). These differences emphasize the importance of personalized treatment planning based on precise anatomic and clinical assessment, guided by current clinical practice guidelines rather than new technologies.

### 2.2 Radiological assessment of EVAR

EVAR involves the placement of a stent graft into the aorta to remove an aneurysmal segment from the systemic blood flow and strengthen the arterial wall. It is predominantly indicated for the treatment of AAA in anatomically suitable patients (4, 12). Despite the challenging anatomical morphology, the latest generation of EVAR devices can address a wide range of complex aortic pathologies, including both the aneurysms and dissections (22).

Preoperative imaging is essential to determine aneurysm size, morphology, and suitability for EVAR. The procedure requires accurate measurement of the proximal and distal landing zones to ensure adequate adherence and fixation of the endograft to healthy arterial tissue and to minimize the risk of device migration and endoleakage (12, 23). Appropriate oversizing is also necessary to ensure effective fixation.

Computed tomography (CT) is the most widely used method for both pre- and post-procedural assessment due to its high spatial resolution and 3D reconstruction capability (22). A noncontrast CT scan can help distinguish calcified thrombi or surgical materials from endoleaks containing contrast material, while CT scans containing contrast material provide a detailed assessment of aneurysm morphology and vascular anatomy (11, 24). Multiphase CT scanning is particularly useful for identifying and classifying endoleaks, one of the most common complications after EVAR (11). 3D evaluation of the aorta is important because even a small increase in the length of the aortic aneurysm can cause a significant increase in its volume. While maximum aortic diameter is currently the gold standard for decision-making, it is not always associated with volumetric expansion. For example, Caradu et al. (8) demonstrated that even a 2 mm increase in diameter can reflect a greater than >5% increase in aneurysm volume. Such observations highlight the value of 3D volumetric assessment in monitoring saccular development However, true volumetric analysis requires advanced segmentation techniques that are time-consuming and not yet widely used in daily practice.

Long-term post-intervention follow-up is crucial for due to the potential for late complications such as endoleaks, saccular expansion, device migration, or delayed rupture (25). Mehta et al. (26) reported approximately 1.5% of EVAR patients experienced delayed rupture at a mean of 29 months after intervention. The Food and Drug Administration (FDA) recommends continuing CTA at 1, 6, and 12 months after EVAR and then annually thereafter indefinitely if no problems are detected (25).

Despite its advantages, CTA has limitations, including radiation exposure, cost, and the risk of contrast-induced nephropathy. Furthermore, segmentation of the aortic wall and thrombus is semi-automatic at most imaging stations. Therefore, fully automated, standardized 3D analysis tools, most often AI-powered, may improve efficiency and consistency in the future but are under evaluation for routine clinical integration (8, 27, 28).

## 3 AI techniques in aortic diseases management

### 3.1 Overview of AI, ML, and DL in aortic pathologies

AI generally refers to computational systems that mimic human cognitive functions, including learning, reasoning, and decision-making (15, 29). In this context, ML refers to algorithms that improve through data visualization, and DL is a specialized subset of ML that uses multilayer neural networks, specifically capable of extracting complex patterns in imaging data (16, 30). In the context of aortic disease, AI models have shown potential in automating diagnostic image segmentation, improving morphological analysis, and supporting outcome prediction (10, 27, 31).

In this review, we use AI as an umbrella term that also encompasses machine learning (ML) and deep learning (DL). DL is the core element of imaging for segmentation and centerline/orthogonal measurements; ML/DL models also support risk prediction related to oversizing and landing zone strategy. We discuss computer vision (CV), where models interpret CT angiography, and extended reality (XR), where planning layers help operationalize device selection (13, 30, 32).

### 3.2 Image-based aortic segmentation and measurement

**Figure 2:**
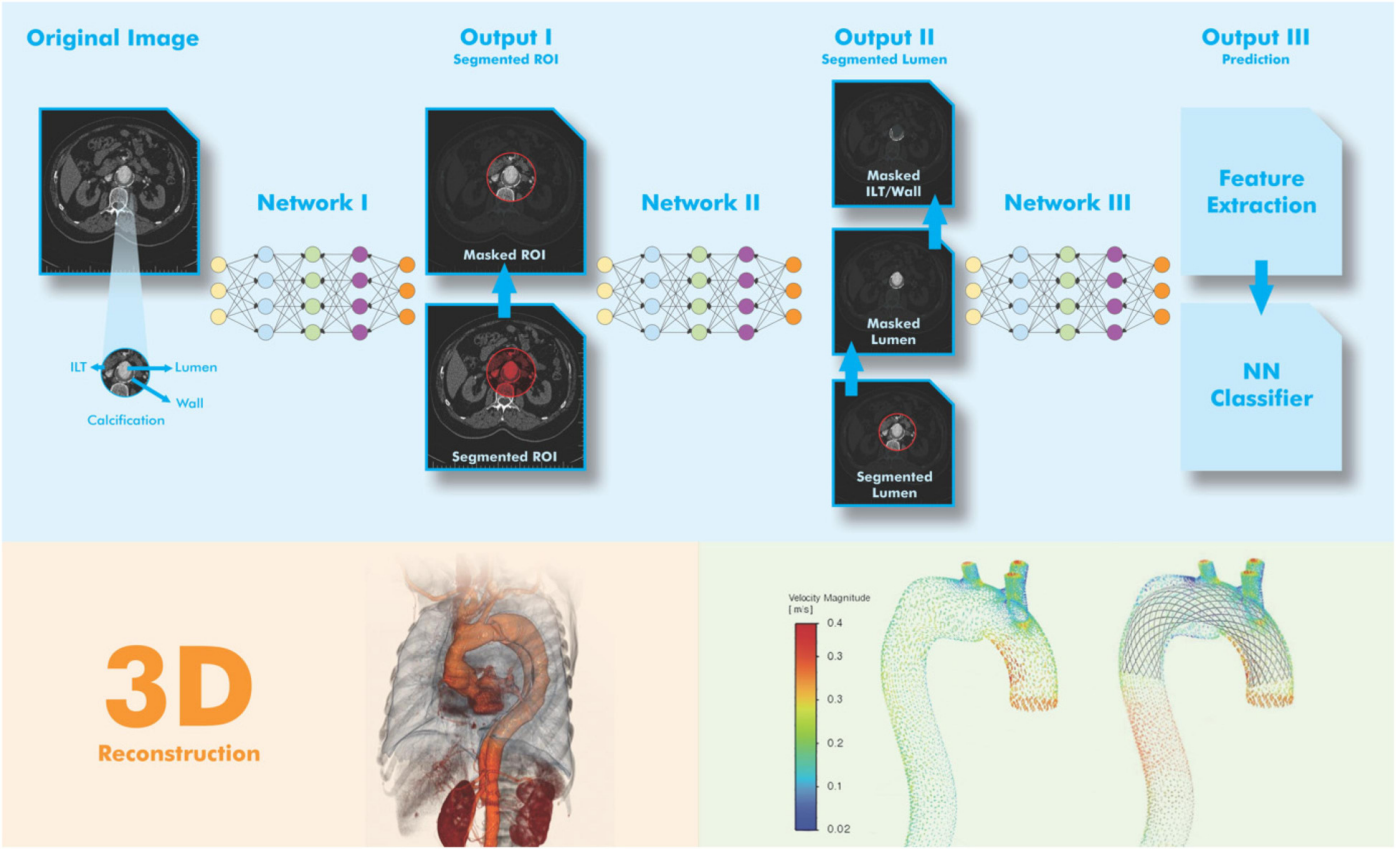
Simplified visual representation of AA segmentation using DL model. The goal of image acquisition (**Step-1**) is to obtain a 3D image of the aorta from a series of DICOM images with 2D cross-sectional slices. In the pre-processing stage (**Step-2**), the image quality is improved and the input for the AI model is standardized. In AI-assisted segmentation (**Step-3**), DL models are trained on thousands of labeled scans. A segmentation mask is applied by marking the pixels corresponding to the aorta and the segmented aorta is analyzed to mark areas with abnormal dilation. In the post-processing stage (**Step-4**), the AI output is cleaned for accuracy. In the reconstruction and visualization stage (**Step-5**), the goal is to display the results in a clear and interpretable way. For this purpose, the aorta is shown with a base color (e.g., blue) and the aneurysm region is shown with a warning color (e.g., red). In the figure, pre-and post-stenting blood flow velocity are figured (right below). Adapted from: “Visual representation of the network architecture and output for AAA tissue segmentation” by Atefeh Abdolmanafi, Arianna Forneris, Randy D. Moore and Elena S. Di Martino, licensed under CC BY 4.0, and “Diagrammatic comparison of the processing framework of machine learning and deep learning” by Nurittin Ardic and Dinc, licensed under CC BY-NC. Drawing with Adobe Creative Suite Package [(Illustrator, version 28.7.1 and Photoshop, version 25.12)].

One of the most studied applications of AI in vascular medicine is the automatic segmentation of abdominal aortic aneurysms (AAA) in CTA datasets (Figure 2). Traditional methods often rely on semi-automated tools that require manual correction, are time-consuming, and operator dependent.

Abdolmanafi et al. (10) developed a DL-based tool that enables highly accurate segmentation of the aneurysmal sac from preoperative CT and allows rapid and reproducible measurement of aortic diameters. Similarly, Adam et al. implemented a fully automated pipeline for maximum diameter assessment before and after EVAR that correlated well with manual measurements (27). These tools can help reduce observer variability and support standardized surveillance, but their clinical use is limited to experimental or retrospective settings (10, 27, 31).

### 3.3 Prediction models and risk stratification

AI has also been applied to predict clinical outcomes after EVAR, including complications such as endoleaks, sac dilation, or reintervention. For example, Karthikesalingam et al. used an artificial neural network to stratify the risk of mortality and reintervention after EVAR and identified high-risk patient profiles based on anatomic and procedural variables (34). More recently, Long et al. proposed a DL-based risk model that integrates procedural and imaging features to predict post-EVAR complications such as type 1 endoleaks and bladder dilation (35). However, these models need to be validated in large, prospective, multicenter cohorts before they can be recommended for routine clinical use.

It is also important to distinguish risk profiling tools from actual treatment guidance. Predictive models can help clinicians adjust surveillance intensity or select ancillary techniques, but they do not replace existing treatment guidelines or procedural decisions.

### 3.4 AI for complication prediction and endoleak classification

Endoleaks are among the most common findings after EVAR. They represent blood flow that continues outside the stent graft lumen but within the aneurysm sac. Endoleaks are classified as types 1–5, with varying clinical outcomes. Importantly, type 2 endoleaks, originating from retrograde flow from branch vessels such as the lumbar or inferior mesenteric arteries, are generally benign and self-limited, while types 1 and 3 endoleaks (inadequate seal or device integrity failure) are associated with a higher risk of sac expansion and rupture (11, 25).

Accurately identifying and classifying endoleaks using CT imaging after EVAR can be challenging due to variations in anatomy, contrast timing, and image quality. AI-based image classification and segmentation tools have been investigated to address these limitations. Long et al. A DL model was developed that integrates procedural variables and imaging features to predict the likelihood of complications, including type 1 endoleaks and sac dilation, with promising accuracy (35). Furthermore, predictive modeling can support early identification of patients at risk for delayed complications such as sac enlargement, migration, or even rupture. Karthikesalingam et al. (34) applied an artificial neural network to stratify the risk of reintervention and mortality after EVAR using large retrospective datasets. While their study demonstrated the applicability of nonlinear models for outcome prediction, external validation remains limited.

Despite these advances, current AI models have not been validated for diagnostic use in real-world clinical settings. Furthermore, risk prediction does not imply an indication for treatment. Decisions regarding reintervention after type 2 endoleaks or sac dilation should adhere to established guidelines and individual patient factors (25).

In summary, AI has the potential to complement post-EVAR surveillance through automated detection and risk prediction; however, it should be viewed as a complement to, and not a substitute for, clinical judgment and guideline-based decision-making.

### 3.5 Limitations and current clinical status

Although preliminary findings are encouraging, the real-world integration of AI models in aortic disease management is limited. Most studies to date are retrospective, single-center, or lack external validation. Regulatory approval for AI-based clinical tools in vascular surgery remains rare (33, 36).

Furthermore, most published models are “black box” in nature; This means that decision-making processes are not interpretable and raises questions about clinical trust and accountability (37, 38). Ethical issues, such as algorithmic bias in underrepresented populations, should also be addressed before wider implementation (39, 40).

## 4 Clinical integration of AI: from workflow support to real-world applications

### 4.1 Workflow and decision support integration

The integration of AI into vascular clinical workflows is an emerging area of research focused on improving the efficiency, accuracy, and consistency of preoperative planning and procedural simulation. These technologies are being developed to support vascular surgeons and interventional radiologists in routine decision-making and patient-specific procedural strategies, rather than replacing clinical judgment.

To delineate how AI supports device selection decisions rather than overall workflow optimization, Table 1 summarizes the key AI components applied to graft sizing, seal site optimization, and procedural planning. The table highlights the decision point targeted by each AI module, the input data, the outputs related to device selection, and the current level of clinical readiness.

**Table 1:** AI components directly supporting device selection decisions in aortic endovascular repair. The table highlights applications that impact sizing, sealing, and procedural planning rather than overall analysis.

Use case (decision point)	Typical inputs	AI output for device selection	Clinical readiness	Representative examples/resources
Automatic centerline extraction and orthogonal measurement for graft sizing/oversizing	CTA DICOM; aortic/iliac geometry	Reproducible diameters and lengths; standard sizing worksheet	CE-marked semi-automatic modules; operator validation required	TeraRecon Intuition™, Siemens syngo.CT Vascular Analysis (35)
Risk prediction of inadequate sealing or type I endoleak guides oversizing and landing zone strategy	Preoperative measurements + anatomic and procedural features	Possibility of endoleak or migration risk; oversizing recommendations	Investigational; retrospective validation	(34, 35)
Intra-procedural augmented intelligence mapping for complex EVAR/TEVAR	Preoperative CTA + live fluoroscopy	Key point registration and site targeting to confirm device configuration	Early clinical reports	(13, 41)
Automated post-EVAR volumetric assessment supports reapplication or extension device planning	Serial CTA scans	Objective measurements of sac and neck evolution to guide reintervention device selection	Investigational use; prospective trials ongoing	(8)

As summarized in Table 1, these targeted modules directly impact the sizing and planning decisions that determine procedure success. The next section summarizes the commercial applications of these functions in clinical practice.

One promising area is the use of AI-enabled software for automated stent graft planning. These tools incorporate preoperative CTA data to assist in selecting appropriate device sizes and configurations based on patient-specific anatomy. Patel et al. have shown that surgical augmented intelligence maps can facilitate more accurate deployment planning, improving radiation safety and contrast utilization during the treatment of complex aortic aneurysms (41). Extended reality (XR) platforms, which refer to technologies that enhance or change our perception of the world by overlaying digital information onto the real world or immersing users in a completely digital environment, are also gaining momentum in simulation-based training and procedural rehearsals, and artificial intelligence modules support real-time anatomical recognition. A novel integration of AI with XR has been described by Samant et al. to optimize planning for high-risk cardiovascular interventions, potentially reducing case time and improving anatomical understanding (13).

Beyond planning, AI-based systems can contribute to intraoperative navigation and real-time decision support. Some platforms can detect anatomical landmarks, identify stent landing zones, and alert operators to discrepancies between planning and live fluoroscopic images. While these applications are experimental, they reflect a shift toward surgical augmented intelligence; these tools enhance clinician performance rather than automating full procedural execution. In the outpatient setting, AI can help classify imaging studies, prioritize complex cases for earlier review, and generate standardized reports using natural language processing (NLP). For example, Fabre et al. (28) proposed a semi-automated system for monitoring aneurysm evolution after EVAR using AI-assisted measurement tracking on serial CTA scans.

However, most of these systems are still in development or pilot stages and have not yet received regulatory approval for widespread clinical use. Additionally, integration with hospital information systems and compatibility with imaging Picture Archiving and Communication Systems (PACS) remain logistical hurdles. However, these innovations highlight the growing importance of AI as a collaborative tool in complex vascular workflows.

The outlined steps in AI-assisted aortic aneurysm intervention are: Patient CTA → Segmentation (AI) → Sizing → Risk stratification (AI) → Device planning (AI-assisted) → Procedure → Postoperative follow-up (AI).

### 4.2 Commercial platforms in practice

While much of the AI innovation in aortic disease management stems from academic research, several commercial AI-powered tools have entered clinical use, particularly in the areas of workflow improvement and triage optimization. These tools primarily serve as decision support systems for preoperative planning, automated imaging analysis, and postoperative surveillance. Box 1 illustrates three representative scenarios where AI directly impacts device selection decisions. However, their integration into routine clinical practice is limited and is typically limited to high-volume centers or pilot programs.

Box 1 **Illustrative device selection scenarios. Illustrative scenarios where AI directly impacts device selection.**

**Case-1: Elective AAA with a short, angulated, thrombus-laden neck.**

A 73-year-old man with a 6.2 cm infrarenal AAA had a proximal neck length of ∼12 mm, an angulation of ∼65°, and mural thrombus/calcification. AI-assisted centerline and orthogonal planes provided reproducible diameters/lengths and a standardized sizing worksheet. A risk model (trained with retrospective data) demonstrated a higher likelihood of inadequate proximal sealing with routine oversizing. The team selected an oversizing window toward the superior end within the instructions for use (IFU), enlarged the planned proximal landing zone, and prepared ancillary maneuvers. Intraoperative augmented intelligence flagging was appropriate; no early type I endoleak was detected. These AI components reduced measurement variability and supported sizing/landing zone decisions; clinical judgment remained primary.

**Case-2: Post-EVAR sac enlargement triggered re-application/extension planning.**

A 70-year-old man demonstrated sac enlargement on surveillance CT at 18 months. Automated volume measurements confirmed greater than 5% sac expansion despite a <2 mm change in maximum diameter, enabling earlier assessment of proximal extension. Standardized evolution curves and centerline-based re-measurement facilitated communication with the sizing team and informed the selection of the extension configuration. In this case, AI-assisted volume measurements and consistent measurement lines supported the decision to proceed, while guideline-based thresholds and imaging review remained decisive.

**Case-3: TEVAR Planning for a type B dissection with a hostile arch.**

A 68-year-old woman with a complicated Type B dissection and tortuous arch required careful landing site strategy. AI-assisted segmentation mapped true/false lumen relationships and branch vessel origins; planning overlays helped evaluate alternative proximal landing sites and projected catheter paths. The final device selection and site strategy were validated through IFU and multidisciplinary discussion. AI tools standardized measurements and visualized tradeoffs but did not replace operator decision-making.

The TeraRecon Intuition™ platform, including the EVAR planning suite, leverages AI to perform 3D centerline extraction from CTA scans, semi-automated aortic measurements, and stent graft sizing. These features aim to reduce interobserver variability and streamline case preparation. While the tool is CE-marked and widely used, it does not operate autonomously and requires user verification during planning steps (42–44).

Similarly, Siemens Healthineers’ syngo. CT Vascular Analysis supports automatic aortic centerline generation, curved planar reformations (CPR), and cross-sectional measurements. The platform also includes modules specific to AAA preoperative workflows, such as stent planning and iliac measurement tools (45–47). While these modules have proven timesaving, independent evaluations are limited to vendor-supported studies.

Viz.ai's Viz Aorta module is one of several FDA-cleared AI tools focused on aortic diseases. It uses deep learning to detect suspicious aortic aneurysms and dissections from CTA scans and prioritize radiology examination. Validation studies have reported 94.2% sensitivity and 97.3% specificity, and the software is currently used in over 850 US hospitals (48, 49). While the tool doesn't recommend treatment, it significantly speeds up triage and facilitates earlier intervention by specialists.

Various ML models have been proposed to predict aneurysm sac growth, postoperative endoleak, and reintervention risk after EVAR (50). For example, Abbas et al. (51) developed a supervised model to predict 1-year sac expansion ≥5 mm with area under the curve (AUC) exceeding 0.90. Similarly, Long et al. (35) and Karthikesalingam et al. (34) have shown that artificial neural networks can outperform traditional statistical models for reintervention risk. However, these models have not yet been validated for clinical use and require prospective validation, multicenter training, and regulatory review.

XR-based platforms, including augmented reality (AR, sub-branch of XR) tools, are increasingly being tested for surgical rehearsal and intraoperative guidance in complex aortic repair. These systems provide comprehensive visualization of patient-specific anatomy, supporting preprocedural planning and team-based coordination. While promising, current AR/XR applications remain primarily research-focused and have not been widely adopted in commercial clinical practice (13).

Another promising platform is PRAE-VAorta (Nurea, France), a fully automated software that provides volumetric and morphological analysis of aneurysm sac and neck evolution after EVAR. The software demonstrated excellent agreement with manually corrected segmentation (Pearson correlation coefficient >0.99; *P* < 0.0001) and significantly shortened segmentation time (2.5 min compared with 22 min per patient; *P* < 0.0001). These features may support earlier detection of adverse sac evolution and improve long-term EVAR surveillance. Although currently in the evaluation phase, PRAE-VAorta exemplifies the type of smart tool ready for clinical integration (8).

To provide a structured overview, Table 2 summarizes the key commercial and research AI applications used in different phases of aortic repair, including their clinical readiness and regulatory status.

**Table 2:** AI tools in aortic repair workflow with clinical readiness.

Workflow stage	AI application	Representative studies	Clinical readiness	Market status	Reference
Preoperative imaging	3D segmentation, centerline extraction	TeraRecon Intuition™ EVAR Suite	CE-marked devices, widely available	Semi-automated graft sizing and planning support	(42–44)
Preoperative imaging	Automated centerline, cross-section	Siemens syngo.CT Vascular Analysis	CE-marked devices, limited independent validation	Supports aortic measuring and graft planning	(45, 47)
Triage and acute care	DL-based CTA detection of aneurysm/dissection	Viz.ai Aorta	FDA approved, in use 850 + centers	Triage flagging, prioritizes radiologist attention	(48, 49)
Intraoperative and preoperative simulation	AR/XR for anatomy visualization and rehearsal	AR simulators (research use)	Academic prototyping only	Team-based rehearsal and guidance	(13)
Postoperative monitoring	ML-based prediction of sac dilatation or endoleak	Custom ML models (academic)	Not approved, retrospective only	Predicts post-EVAR outcomes	(34, 35, 51)
Postoperative monitoring	Fully automated volumetric and morphological analysis of sac and neck evolution after EVAR	PRAE-VAorta	Feasibility studies; excellent agreement with manual segmentation (r > 0.99)	Research use, under evaluation	(8)

## 5 Model validation, refinement, and explainability

### 5.1 Data quality and validation challenges

The development and application of AI models in aortic diseases (especially for EVAR planning, complication prediction, and postoperative surveillance) is limited by data availability and model generalizability issues. Most existing studies rely on single-center, retrospective datasets with limited demographic and geographic diversity. This creates a risk of overfitting and limits clinical applicability to broader patient populations (38, 52).

Most importantly, few models undergo external validation. Because rigorous testing on independent datasets from different institutions or populations is lacking, reported performance metrics (e.g., accuracy, AUC) during development are often overly optimistic. For example, many aneurysm growth prediction models have demonstrated promising AUCs (>0.90) during internal testing but have not yet been evaluated in prospective or real-time clinical settings (35, 51).

Another common challenge is the heterogeneity in imaging protocols and annotation standards. CTA quality, phase timing, and slice thickness vary across institutions, hindering model reproducibility. Annotated ground truth data (especially for segmentation tasks such as aneurysm sac evolution or endoleak classification) is often manually curated, leading to interobserver variability and inconsistent labeling (53, 54).

Furthermore, publicly available datasets in this field remain insufficient. Compared to fields such as radiology or dermatology, aortic imaging lacks large, diverse, and standardized data repositories to support open benchmarking. This limits transparency and makes it difficult to compare model performance across studies (55, 56).

To meet regulatory and clinical standards, future research should prioritize multicenter data collection, standardized disclosure protocols, external validation in temporally and demographically diverse cohorts, publication of negative results and failure cases to increase transparency. Without addressing these fundamental data issues, AI models for aortic care risk remaining experimental, despite strong technical performance in controlled settings. Lack of external validation for device selection leads to uncertain reliability of oversizing windows and landing zone recommendations across scanners and centers.

### 5.2 Explainability and clinical confidence

Explainability is a critical barrier to the clinical adoption of AI in vascular care. While DL models can outperform traditional statistical techniques in classification and segmentation tasks, they are often perceived by clinicians as “black boxes”, producing results without a clear understanding of the reasoning behind decisions (37, 38). This uncertainty creates challenges in establishing confidence, especially when model predictions conflict with clinical judgment.

To address this gap, explainable artificial intelligence (XAI) approaches, such as saliency maps, attention mechanisms, and decision trees, are being explored to make model outputs more interpretable. For example, highlighting which features on a CTA scan contribute most to aneurysm classification can help clinicians assess whether the model's logic aligns with anatomical expectations (54). Despite these efforts, studies show that many existing XAI techniques are inherently unreliable and can create bias or false confidence in system outputs. Furthermore, clinicians’ preferences for visual and textual explanations vary, and integration into clinical systems should consider user-centered design (38, 56).

Regulatory bodies such as the FDA and EMA are now emphasizing transparency and traceability in AI/ML-based software (SaMS) used as medical devices. To comply with evolving standards, developers are required to document model behavior, training data lineage, and expected performance across patient subgroups (36). Explainability must indicate which anatomical features (e.g., neck diameter, thrombus burden, angulation) drive a risk estimate or sizing recommendation; without this, clinicians cannot rely on AI outputs that oversize or alter landing zone plans.

### 5.3 Regulatory issues

The use of AI in aortic disease management, particularly for clinical decision support, image interpretation, and outcome prediction, requires careful consideration of regulatory frameworks. In the US, the FDA regulates AI tools used for clinical purposes under the category of Software as a Medical Device (SaMD). Recent efforts, such as the FDA's “Digital Health Software Pre-Certification Program” and Good Machine Learning Practices (GMLP) guidance, reflect an evolving approach to safety assessment. Europe implements similar oversight through the European Medicines Agency (EMA) and the Medical Device Regulation (MDR), emphasizing CE marking, performance verification, and post-market surveillance. However, regulatory guidance for continuously learning AI models (those that adapt over time) is still under development, creating challenges for clinical application (36).

A key regulatory concern is bias and fairness. Studies have shown that AI models trained on homogeneous datasets can underperform in underrepresented populations, exacerbating health inequalities (39, 49). To mitigate these risks, developers should ensure demographic diversity in training datasets, include bias checks, and stratify model performance by subgroup.

Transparency is another critical issue. Regulatory bodies now require developers to document not only the algorithm architecture and training performance, but also the clinical intended use, limitations, and explainability features. There are growing expectations that models used in aortic care must undergo not only technical validation but also clinical utility evaluations, including prospective trials or real-world evidence studies. Both the FDA and EMA are moving toward a total product lifecycle strategy for AI-based medical software; this approach ensures that performance remains traceable and auditable while supporting adaptive learning. In the US, the FDA's Draft Guidance on AI-Enabled Device Software Functions outlines a Predetermined Change Control Plan (PCCP) framework that allows for post-distribution algorithm updates while maintaining regulatory oversight (57). Meanwhile, the EMA published its Reflection Paper on the use of AI in the medical product lifecycle, advocating for a risk-based lifecycle management model and expecting developers to monitor AI performance at all stages, from development and distribution to post-authorization (58). Regulators are increasingly expecting the intended use specified at the decision stage (e.g., supports graft sizing within the IFU), with performance stratified by anatomy and scanner protocol, which directly relates to EVAR/TEVAR device selection.

As regulations mature, interdisciplinary collaboration among clinicians, AI developers, and regulatory experts will be vital to safely integrating AI into aortic care pathways.

## 6 Future directions and ethical considerations

To fully benefit from these technological advances, current challenges must be addressed. Integrating AI into existing surgical planning software as part of real-time decision support systems, can reduce procedure times and improve outcomes by offering real-time recommendations for stent graft selection.

As machine learning models continue to mature, future applications in EVAR and TEVAR workflows are expected to transition from passive decision support systems to dynamic and adaptive platforms. These systems can combine real-time hemodynamic data, multimodal imaging, and intraoperative feedback to instantly optimize treatment strategies. The integration of federated learning, which enables model training across multiple institutions without data sharing, offers a promising avenue for developing robust and generalizable models while preserving patient privacy (59). Future clinical settings could utilize these tools for personalized graft design or the automatic identification of high-risk anatomical variants.

To ensure clinical robustness and generalizability, future AI systems must be trained and validated using heterogeneous, multicenter datasets under standardized protocols. Initiatives such as the Medical Imaging and Data Resource Center (MIDRC) in the United States and the European Health Data Space (EHDS) in the European Union aim to support this effort by promoting federated learning, harmonized data sharing, and secure access across institutions and jurisdictions (59–61). Additionally, academia-industry collaborations should prioritize not only technical performance but also patient-centered outcomes and equity. It is crucial to ensure that AI development addresses global health inequalities, particularly for underrepresented populations that may exhibit different aortic disease phenotypes.

The adoption of AI in vascular surgery raises important ethical issues related to bias, transparency, and patient autonomy. The lack of interpretability in deep learning models can limit clinician trust, especially when predictions contradict established guidelines. XAI methods are being developed to address “black box” nature of AI, but most are limited to research settings (62). Interactive visualizations, such as attention maps, provide insights into model decisions (37, 38).

From a legal perspective, questions of liability in the event of AI-induced misdiagnosis or planning errors remain unresolved. Institutions should implement clear policies regarding oversight, documentation, and escalation protocols when AI tools are deployed in clinical pathways. In parallel, regulators are exploring risk-based classifications and real-time monitoring frameworks to ensure safety without hindering innovation (63).

In conclusion, the integration of AI into intravascular device selection for aortic disease is an area of active research. From preoperative segmentation and simulation to intraoperative guidance and postoperative surveillance, AI tools can offer supporting capabilities to increase precision and reduce variability in complex cases. However, current endovascular techniques currently deliver excellent results, and the added value of AI remains to be proven through large-scale validation and clinical trials. AI-powered approaches should be viewed as complements, not replacements, of established guideline-based care. The widespread clinical implementation landscape will depend on overcoming challenges related to data quality, explainability, interoperability, and regulatory approval. Continued interdisciplinary collaboration is essential to ensure the safe, equitable, and clinically meaningful development and implementation of these tools. Near-term priority is to prospectively test modules that standardize centerline/orthogonal measurements and calibrate oversizing against endoleak risk.
